# Fibrin promotes oxidative stress and neuronal loss in traumatic brain injury via innate immune activation

**DOI:** 10.1186/s12974-024-03092-w

**Published:** 2024-04-15

**Authors:** Terry Dean, Andrew S. Mendiola, Zhaoqi Yan, Rosa Meza-Acevedo, Belinda Cabriga, Katerina Akassoglou, Jae Kyu Ryu

**Affiliations:** 1 Gladstone Institute for Neurological Disease, San Francisco, CA USA; 2 Center for Neurovascular Brain Immunology at Gladstone, University of California San Francisco, San Francisco, CA USA; 3 Department of Neurology, Weill Institute for Neurosciences, University of California San Francisco, San Francisco, CA USA; 4 Present Address: Center for Neuroscience Research, Children’s National Hospital, Washington, DC USA; 5 Present Address: Department of Pharmacology, University of California San Diego, La Jolla, CA USA

## Abstract

**Background:**

Traumatic brain injury (TBI) causes significant blood-brain barrier (BBB) breakdown, resulting in the extravasation of blood proteins into the brain. The impact of blood proteins, especially fibrinogen, on inflammation and neurodegeneration post-TBI is not fully understood, highlighting a critical gap in our comprehension of TBI pathology and its connection to innate immune activation.

**Methods:**

We combined vascular casting with 3D imaging of solvent-cleared organs (uDISCO) to study the spatial distribution of the blood coagulation protein fibrinogen in large, intact brain volumes and assessed the temporal regulation of the fibrin(ogen) deposition by immunohistochemistry in a murine model of TBI. Fibrin(ogen) deposition and innate immune cell markers were co-localized by immunohistochemistry in mouse and human brains after TBI. We assessed the role of fibrinogen in TBI using unbiased transcriptomics, flow cytometry and immunohistochemistry for innate immune and neuronal markers in *Fgg*^*γ390–396A*^ knock-in mice, which express a mutant fibrinogen that retains normal clotting function, but lacks the γ390–396 binding motif to CD11b/CD18 integrin receptor.

**Results:**

We show that cerebral fibrinogen deposits were associated with activated innate immune cells in both human and murine TBI. Genetic elimination of fibrin-CD11b interaction reduced peripheral monocyte recruitment and the activation of inflammatory and reactive oxygen species (ROS) gene pathways in microglia and macrophages after TBI. Blockade of the fibrin-CD11b interaction was also protective from oxidative stress damage and cortical loss after TBI.

**Conclusions:**

These data suggest that fibrinogen is a regulator of innate immune activation and neurodegeneration in TBI. Abrogating post-injury neuroinflammation by selective blockade of fibrin’s inflammatory functions may have implications for long-term neurologic recovery following brain trauma.

**Supplementary Information:**

The online version contains supplementary material available at 10.1186/s12974-024-03092-w.

## Introduction

Traumatic brain injury (TBI) is a leading cause of death and disability worldwide, however, effective targeted therapies are notably lacking [1]. TBI is characterized by significant disruption of the blood-brain barrier (BBB), neuroinflammation, and neuronal cell loss [2]. Although the clinical focus of BBB disruption following neurotrauma has traditionally been on its contribution to cerebral edema in the acute setting [3, 4], recent evidence suggests that BBB dysfunction may persist for years after injury [5]. In other neurologic diseases, including Alzheimer’s disease (AD) and multiple sclerosis (MS) [6], the extravasation of blood products into the brain following BBB disruption contributes to neuroinflammation and neuron loss. However, whether a similar mechanism is involved in TBI is unknown.

Fibrinogen is an abundant blood protein that plays an essential role in coagulation. Upon leakage into the brain, fibrinogen is converted to fibrin, which accumulates in the brain parenchyma [6]. Fibrinogen conversion to fibrin exposes a cryptic epitope on the fibrin γ-chain, which can bind to the CD11b/CD18 integrin receptor on microglia and infiltrating macrophages [6–12]. Fibrin-CD11b/CD18 interaction induces signaling within the immune cells that promotes a unique pro-inflammatory transcriptional signature, release of reactive oxygen species (ROS) and leukocyte-attracting chemokines causally linked to neurodegeneration in models of AD and MS [8–13]. Because TBI also induces significant BBB disruption and fibrin deposition in the CNS [5, 14], and innate immune activation contributes to TBI-induced neuropathology [15, 16], we hypothesized that fibrin-CD11b/CD18 signaling may be a critical molecular mediator linking BBB permeability and neuroinflammation in TBI.

Here, we show that fibrin promotes immune-mediated neurodegeneration in TBI. Fibrin deposits were associated with innate immune inflammatory markers in human TBI samples and in a murine model of TBI. We then tested the effects of genetic blockade of fibrin’s inflammatory function using *Fgg*^*γ390–396A*^ mice, which express mutant fibrinogen that retains normal clotting function, but lacks the γ390–396 binding motif to CD11b/CD18 integrin receptor [7, 8]. *Fgg*^*γ390–396A*^ mice had reduced activation of microglia and monocyte inflammatory pathways following TBI than control mice, resulting in decreased macrophage recruitment, oxidative damage and neuronal loss. Together, these data suggest that fibrin interaction with CD11b/CD18 receptor could be a novel therapeutic target for traumatic injuries that otherwise lack targeted therapies.

## Materials and methods

### Animals

All mouse experiments were performed in the C57BL/6 genetic background using protocols approved by the Committee of Animal Research at the University of California, San Francisco, and in accordance with the National Institutes of Health guidelines. The program is accredited by the Association for the Assessment and Accreditation of Laboratory Animal Care. C57BL/6 mice, B6.129P2-*Cx3cr1*^*tm1Litt*^/J (“*Cx3cr1*^GFP/GFP^”) mice [17], and B6.129-*Ccr2*^*tm2.1lfc*^/J (“*Ccr2*^RFP/RFP^”) mice [18] were purchased from the Jackson Laboratory for controlled cortical impact (CCI) experiments. *Cx3cr1*^GFP/GFP^ and *Ccr2*^RFP/RFP^ mice were crossed to yield *Cx3cr1*^GFP/+^*Ccr2*^RFP/+^ progeny. *Fgg*^*γ390–396A*^ mice were obtained from Dr. Jay Degen (University of Cincinnati, OH, USA) [7]. Males aged 17–24 weeks were used for all experiments. Mice were housed in groups of a maximum of five mice per cage under standard vivarium conditions with access to standard laboratory chow and water *ad libitum* and a 12-h light: dark cycle. All animal protocols were approved by the Committee of Animal Research at the University of California, San Francisco, and in accordance with the National Institutes of Health Guidelines.

### Human tissue and immunohistochemistry

Human brain samples were obtained via the NIH NeuroBioBank. The “acute TBI” sample came from a 32-year-old male who had suffered a gunshot wound to the head within 24 h before autopsy. The sample arrived flash frozen, was processed and sectioned by the Gladstone Histology and Light Microscopy Core, fixed for 5 min in ice-cold acetone, and then subjected to immunohistochemistry. The “chronic TBI” sample came from a 21-year-old female who had survived for one year after blunt head trauma. The sample arrived formalin fixed, was processed and sectioned by the UCSF Biorepository and Tissue Biomarker Technology Core, deparafinized, rehydrated, and subjected to heat-mediated antigen retrieval (Target Retrieval Solution, Low pH, DAKO) for 1 h at 95 °C before proceeding with immunohistochemistry. Immunohistochemistry was performed as previously described [19] with the following antibodies: 1:500 mouse monoclonal anti-fibrinogen Ab [Abcam], 1:500 rabbit monoclonal anti-CD68 Ab [Abcam], 1:400 rabbit polyclonal anti-Iba1 [WAKO], 1:400 mouse monoclonal anti-MRP14 Ab [DAKO]. Images were acquired with an Axioplan II epifluorescence microscope (Carl Zeiss) equipped with dry Plan-Neofluar objectives (10 × 0.3 NA, 20 × 0.5 NA, or 40 × 0.75 NA), an Axiocam HrcCCD camera, and the Axiovision image analysis software. For each sample, high-powered fields were taken from a single slide with immune markers and fibrinogen immunoreactivity as histological landmarks.

### Murine controlled cortical impact (CCI) model of TBI

CCI was performed as described previously [16], with modifications. Mice were anesthetized with buprenorphine (∼ 0.05 mg/kg) and ∼ 2% inhaled isoflurane through a non-rebreathing nose cone on a heating pad at 37 °C. After fixation with non-traumatic ear bars to a stereotactic frame (Kopf), eyes were provided with lubricant and the scalp was shaved and sterilized with betadine solution. Using sterile tip technique, a midline incision was made, and a ∼ 3.5 mm craniectomy was performed using an electric drill, with center point at 2.0 mm posterior and 2.0 mm lateral to bregma, taking care to not breach the dura. An electromagnetic cortical impactor (Hatteras, NC) delivered the injury with the following specifications: depth 0.9 mm, velocity 2.5 m/s, duration 500 ms. Afterwards, the scalp was stapled and the mouse was left to recover in a warmed cage (37 °C) until resumption of normal behaviors when it could rejoin its littermates. Sham controls underwent anesthetic induction, scalp incision, and craniotomy, but did not undergo cortical impact.

### Mouse tissue immunohistochemistry

Dissection of brains and immunohistochemistry were performed as previously described [9], with following primary antibodies: rabbit polyclonal anti-fibrinogen (J. L. Degen, Cincinnati Children’s Hospital Medical Center), mouse monoclonal anti-NeuN (Millipore), and mouse monoclonal anti-4-Hydroxy-2-nonenal (HNE, Alpha Diagnostic International). Images were acquired with a BIOREVO BZ-9000 inverted fluorescence microscope (Keyence) equipped with a Nikon CFI 60 series infinite optical system and Keyence imaging software or an Olympus FluoView 1000 confocal microscope with water-immersed objectives (20 × 0.5 NA or 40 × 0.64 NA). ImageJ (NIH) was used for image analysis. Depending on the staining, quantification was performed on thresholded, binary images or counting of cells by researchers blind to the mouse genotype. Homozygous *Fgg*^*γ390–396A*^ mice were compared to C57BL/6 mice as non-transgenic wildtype colony controls for the CCI histology experiments.

For fibrin(ogen) staining, a region of interest (ROI) was established for each slice consisting of all cortex within 250 μm of the impacted edge (for TBI group) or craniotomy (sham). Relative intensity of fibrinogen within this region was compared to a 250 μm by 250 μm region of the contralateral, uninjured cortex. Three slices were averaged per mouse, with *n* = 4–7 mice per timepoint. Statistics performed with one-way ANOVA, with Dunnett’s multiple comparisons test to compare each timepoint to sham mice.

For quantification of fluorescent cells in *Cx3cr1*^GFP/+^*Ccr2*^RFP/+^, 150 × 150 μm were captured from a larger image of cortex containing the lesioned edge: ROI were obtained either adjacent to the lesion (i.e. an ROI with a complete border at the lesion edge and extending into cortex) or remote from the lesion (i.e. an ROI located at the furthest extent ∼ 300 μm away from injury). For each ROI, the fibrinogen intensity was quantified separately from the cell counting, where the scorer was blinded to location or fibrinogen content. Upon cell counting, CX3CR1 positive cells were further classified as to whether they were morphologically “amoeboid” (or “activated”): cells that lacked an obvious ramified/stellate morphology, instead demonstrating prominent, rounded cell somata with very few, shortened processes. Three adjacent and three remote ROIs were selected per mouse. These data were analyzed via linear regression or unpaired student’s t-test, as detailed.

For quantification of SMI-32 staining, images were acquired from an ROI consisting of cortex within 250 μm of the affected edge with an Axioplan II epifluorescence microscope (Zeiss) equipped with dry Plan-Neofluar objectives (10 × 0.3 NA, 20 × 0.5 NA, or 40 × 0.75 NA). Quantification of SMI-32 immunoreactive area was performed on thresholded images using ImageJ. Three sections were selected per mouse, with *n* = 4 mice. The relationship between fibrinogen and SMI-32 was determined by linear regression analysis. To measure oxidative damage levels, the CA1 region in the ipsilateral side was immunostained for 4-hydroxynoneal (4-HNE) protein adducts, a marker of lipid peroxidation. The 4-HNE immunoreactive area was quantified on thresholded images utilizing ImageJ software.

For quantification of cortical loss, immunostaining for NeuN was used to identify cortex in coronal slices at identical positions from bregma (i.e. -1.9 to -2.7 mm). The contralateral “cortical fraction” was calculated as a percent area of the depicted hemisphere that was designated as cortex. For ipsilateral cortex, an outline of a non-deformed hemisphere was produced (using the contralateral cortex as a guide); cortical fraction was calculated as percent area of the of the outlined cortex that was designated as remaining cortex.

### Cerebral vessel casting and uDISCO

At the time of CCI, wild-type mice (*n* = 3) were injected with fluorescently tagged Alexa^647^-fibrinogen (Invitrogen). At 2 days following injury, mice underwent vessel casting and perfusion. Briefly, mice were injected with 2.5% tribromoethanol followed by transcardial perfusion with 20 mL of PBS and 20 mL of 4% PFA in PBS. This was followed by perfusion with 10 mL of fluorescent gel perfusate (2% w/v PBS porcine skin gelatin type A with 100 mg fluorescein 2MDa dextran) at 40 °C, followed by rapid body cooling, as previously described [20]. Brain and skull were kept intact as they underwent post-fixation in 4% PFA overnight at 4 °C. Mouse brain tissue was then processed using the uDISCO brain clearing protocol as previously described [21] with BABB-D15 refractive index matching solution. Samples were imaged using a Nikon A-100 light-sheet microscope equipped with a Vortran 4-line laser launch, an Andor Zyla 5.5 camera, and a 2x/0.2 AZ Plan Apo objective lens to obtain large-volume images. Image processing and analysis were performed with Imaris and ImageJ.

### Fluorescence-activated cell sorting (FACS)

Following TBI experiments, animals were perfused with 4 °C DPBS and a 1 mm tissue block spanning the lesion sites of each mouse was isolated. To increase cell yield for RNA-seq analysis, two mouse brain tissue samples per genotype and timepoint were randomly pooled together for each biological replicate. Single cell suspensions were prepared using the adult brain dissociation kit (Miltenyi Biotec) as described [22]. Samples were then treated with Fc-block in DPBS supplemented with 0.2% BSA (Thermo Fischer Scientific) for 5 min at 4 °C, then incubated with primary antibodies for 30 min at 4 °C. The following primary antibodies were used at 1:200 dilution and purchased from Biolegend: CD11b (M1/70) conjugated to APC-Cy7 and CD45 (30-F11) conjugated to 488. Samples were incubated with Sytox blue live/dead stain for 10 min prior to sorting as previously described [13]. FACS of 40,000 live microglia with CD45^low^CD11b^+^ expression or 25,000 live infiltrating myeloid cells with CD45^hi^CD11b^+^ expression were sorted directly into tubes containing RLT plus lysis buffer (Qiagen) supplemented with 1% 2-mercaptoethanol and 0.25% reagent DX (Qiagen) using FACSAria II with BD FACSDiva v8 software. Based on our prior bulk RNA-seq studies [13, 22], samples with > 20,000 sorted cells were used for RNA-seq library preparation. Microglia were sorted 1d and 7d after injury. Myeloid cells were only sorted at 1d after injury as fewer than 20,000 cells were detected at day 7. Gating strategy: first gate, cells based on SSC-A and FSC-A size; second gate, doublet discrimination using FSC-H and FSC-W parameters; third gate, sytox blue negative live cells; final gate, either CD45^lo^CD11b^+^ or CD45^hi^CD11b^+^. Lysates were frozen and stored at -80 °C until processed for RNA-seq.

### Bulk RNA sequencing (RNA-seq) and data analysis

RNA was isolated from microglia or macrophage lysates using the RNAeasy micro kit without modification (Qiagen). RNA quality control (QC) was determined by Bioanalyzer pico chip analysis (Agilent) and all samples with RNA integrity number > 8.0 were used for library preparation. cDNA libraries were generated using the Ovation RNA-seq System V2 low input kit following manufacturer’s instructions (NuGEN). Libraries were QC checked by KAPA qPCR (Roche) and Bioanalyzer DNA chip analysis (Agilent). All 21 libraries were equimolar pooled and sequenced across three lanes on Hiseq4000 with paired-end 100 base pairs (Illumnia). The median sequencing depth was ∼ 63 million reads per sample. FASTQ files were processed using the open-access Nextflow RNA-seq pipeline [23] using nextflow v20.12.0-edge in Singularity with default nf-core/rnaseq v3.0 parameters for paired-end FASTQ files. The following packages were used: Bioconductor-summarized experiment v1.20.0; bedtools v2.29.2; deseq2 v 1.28.0; dupradar v1.18.0; fastqc v0.11.9; picard v2.23.9; preseq v2.0.3; rseqc v3.0.1; salmon v.1.4.0; samtools v1.10; star v2.6.1d; stringtie v2.1.4; subread 2.0.1, trimgalore v0.6.6 and ucsc v377. FASTQ files were mapped to GRCm38 genome. Gene analyses was performed in R v4.2.0 using DeSeq2 v1.36.0 on the salmon.merged.gene_counts_scaled file produced by Nextflow. Reads with fewer than 5 counts per gene across all replicates were filtered out. For differential gene analysis the *results* function in DeSeq2 was used with contrast to test between two genotypes and timepoints of interest. The data were then filtered for significance using abs(log_2_FC) > 1 and adjusted *P* value < 0.05 unless otherwise stated. Unbiased KEGG analysis was performed using clusterProfiler and enrichplot with default parameters and pvalueCutoff set to 0.1.

### Statistical analysis

Data were analyzed with Graphpad Prism with statistical testing as described in the text. Data are expressed as the mean ± s.e.m. All mice survived until the end of the study, and all of the data were analyzed. Mice were assigned and coded in a blinded manner for group allocation and subsequent data collection after the analysis. Statistical significance was determined using a non-parametric, two-sided Mann-Whitney test, or one-way or two-way analysis of variance, followed by Dunnett’s or Bonferroni’s multiple comparisons test. All attempts at replication following the protocols described in the methods were successful. For IHC experiments, image acquisition and quantification were performed by observers blinded to experimental conditions. Images were quantified independently by blinded observers. Sample sizes were determined by prior studies rather than statistical approaches.

## Results

### Fibrinogen extravasation and neuroinflammation in human and murine traumatic brain injury

**Fig. 1:**
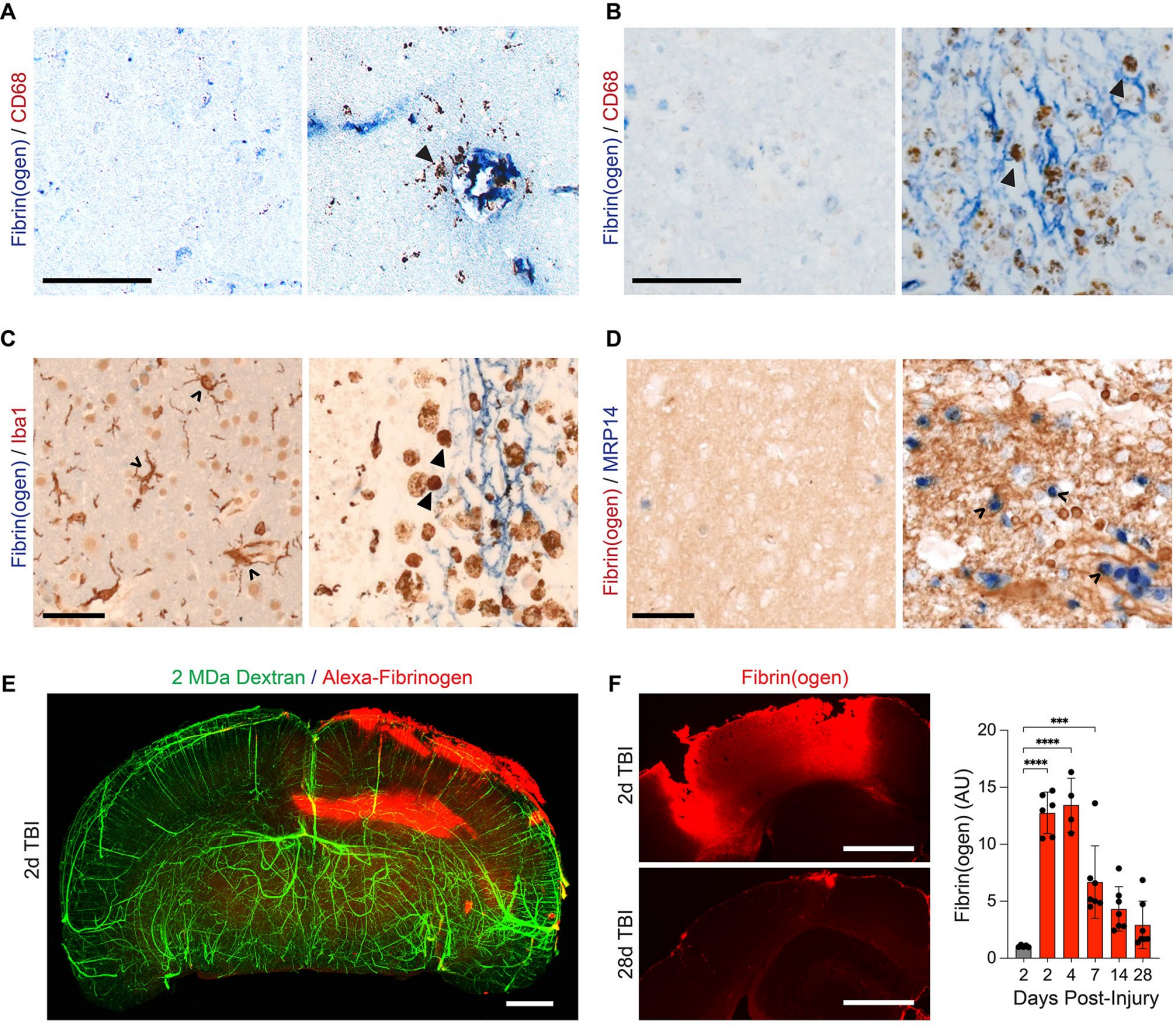
Fibrin(ogen) deposition in human and mice TBI. (**A, B**) Images from human brain tissue less than one day after TBI (A) and one year after injury (B), labeled for fibrin(ogen) (blue) and CD68 (brown). CD68 + cells (Δ) are present in areas with extravasated fibrin(ogen) (right panel), while not detected in areas without fibrin(ogen) (left panel). Scale bars, 100 μm. (**C**) Images from human brain one year after TBI, stained for fibrin(ogen) (blue) and Iba1 (brown). Amoeboid microglia (Δ) are present adjacent to parenchymal fibrin(ogen) deposits (right panel), while they are not detected in areas without fibrin(ogen) (left panel). Scale bar, 50 μm. (**D**) Images from human brain one year after TBI, stained for fibrin(ogen) (brown) and monocytes (MRP14, blue). MRP14^+^ monocytes (^) are present in areas adjacent to dense fibrin(ogen) deposits (brown) (right panel), while they are not detected in areas without fibrin(ogen) (left panel). Scale bar, 50 μm. (**E**) 3-D volume projection of a coronal view of a mouse brain with 2 MDa Dextran-casted vessels (green) and Alexa^647^-fibrinogen (red) 2-days after CCI. Representative of *n* = 3 mice. Scale bar, 1 mm. (**F**) (Left) Representative coronal images of peri-lesional mouse cortex stained for fibrin(ogen) (red) at 2 and 28 days post-injury (DPI). Scale bar, 500 μm. (Right) Quantification of fibrin(ogen) intensity in peri-lesional cortex relative to uninjured, contralateral cortex at multiple timepoints after CCI (red) or sham surgery (gray). Data are presented as mean ± s.e.m.; ***P < 0.001, ****P < 0.0001 determined by one-way ANOVA with Dunnett’s test for multiple comparisons; *n* = 4–7 mice per timepoint, each point is one mouse)

To examine whether fibrinogen extravasation occurs in human TBI and is associated with neuroinflammation, we analyzed autopsy samples from two patients with either acute or chronic TBI. Fibrin(ogen) staining was apparent in both samples associated with CD68^+^ cells (Fig. 1A, B), consistent with innate immune cell recruitment and activation. These amoeboid cells also expressed Iba1 (Fig. 1C) and MRP14, two markers associated with activated microglia and infiltrating monocytes [24, 25] (Fig. 1D), similar to findings in other neurologic diseases (e.g. AD, MS) [6, 26].

To evaluate the relationship between parenchymal fibrinogen deposits and innate immune activation following neurotrauma, we employed an open head CCI murine model of TBI. By combining vascular casting [27] with 3D imaging of solvent-cleared organs (uDISCO) at the peak of post-TBI cerebral edema (i.e. 2 days [28]), we studied the spatial distribution of fluorescently labeled fibrinogen (Alexa-fibrinogen) extravasation in large, intact brain volumes. CCI induced widespread Alexa-fibrinogen extravasation in brain areas ipsilateral to the injury, including the cortex, corpus callosum, and hippocampus (Fig. 1E, Video S1). Furthermore, CCI induced persistent cortical fibrin(ogen) immunoreactivity that peaked at 4 days after injury followed by a gradual decrease over subsequent weeks towards the level found in the uninjured, sham condition (Fig. 1F).

### Spatial dynamics of fibrinogen interaction with CNS innate immunity induces post-TBI inflammation

**Fig. 2:**
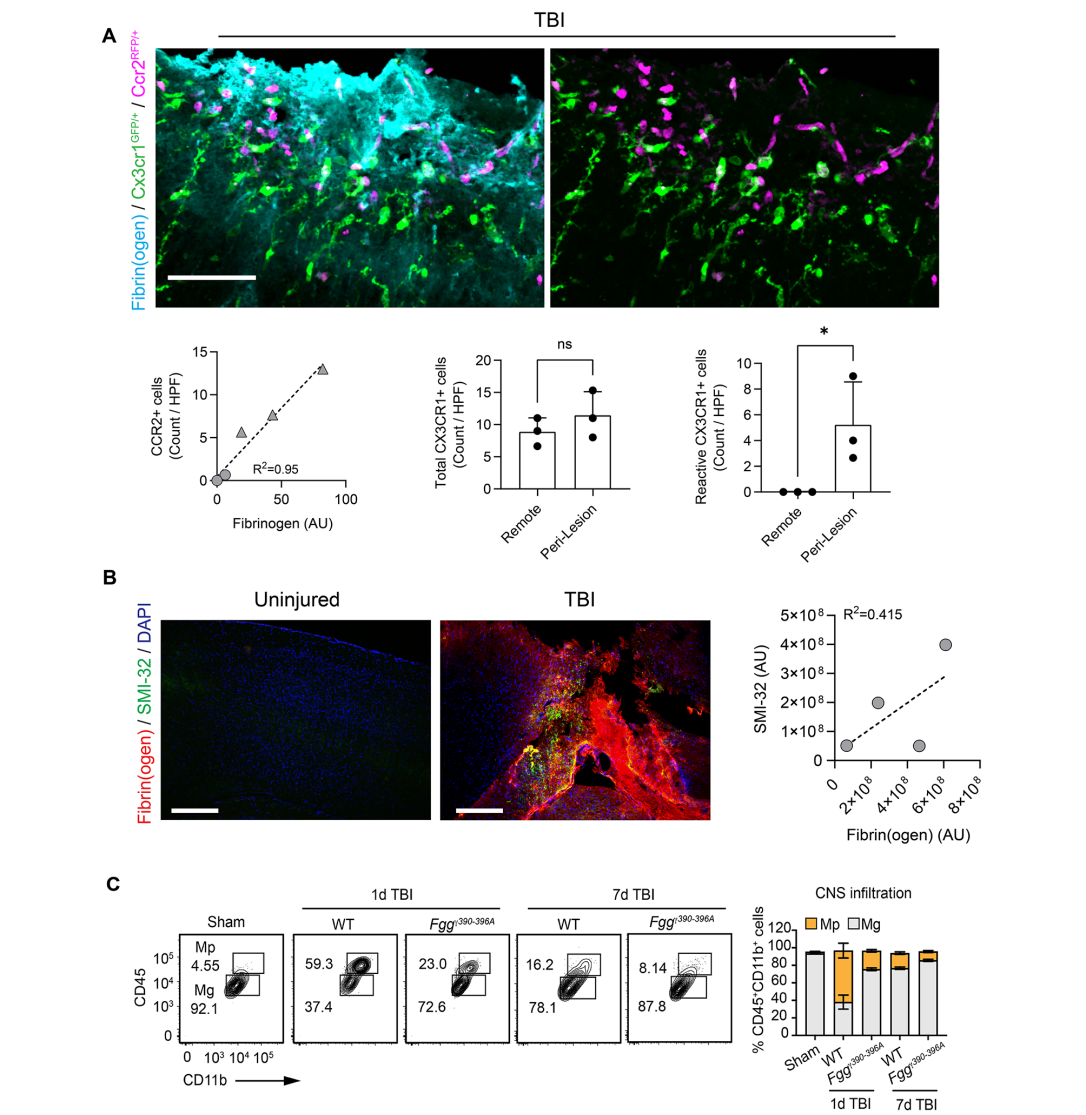
Fibrin regulates microglia activation and myeloid cell infiltration in TBI. (**A**) (Top) Superimposed images of cortex from a *Cx3cr1*^GFP/+^*Ccr2*^RFP/+^ mouse at 2 days post-injury, stained for fibrin(ogen) (cyan), with amoeboid, “reactive” GFP^+^ microglia (green) and RFP^+^-expressing macrophages (magenta). Scale bar, 50 μm. (Below, left) Quantification of fibrin(ogen) staining intensity (X-axis) with the number of CCR2^+^ cells per field. Three peri-lesional regions of interest (triangles) and three remote regions 300 μm from the lesion site (circles) were analyzed per mouse (*n* = 3 mice; linear regression with goodness of fit). Each symbol represents the data for an individual mouse. (below, mid and right) Quantification of total and amoeboid CX3CR1^+^ cells in regions of interest that were lesional or peri-lesional areas from injury 1 day after CCI. Data from *n* = 3 mice (mean ± s.e.m.). HPF, high-power field. ns, not significant. *P < 0.05 by two-tailed Mann-Whitney test. (**B**) Confocal images from mouse cortex 2-days post-CCI, stained for fibrin(ogen) (red), SMI-32 (green), and DAPI (blue). Scale bar, 400 μm. (Right) Scatter plot of fibrin(ogen) (X-axis) and SMI-32 (Y-axis) intensities (*n* = 4 mice; linear regression with goodness of fit). Each circle represents the data for an individual mouse. AU, arbitrary units. **(C)** Representative flow cytometry contour plots of microglia (CD45^lo^CD11b^+^; Mg) and infiltrated myeloid cells (CD45^hi^CD11b^+^; Mp) in brains of TBI 1d and 7d after injury or sham mice. Flow cytometry cell quantification is shown. Data are presented as mean ± s.e.m.; *n* = 5 mice (sham), 4 mice (1d TBI WT), 4 mice (1d TBI *Fgg*^*γ390–396A*^), 3 mice (7d TBI WT), and 3 mice (7d TBI *Fgg*^*γ390–396A*^)

We next evaluated the spatial relationship between parenchymal fibrinogen deposits and innate immune activation following CCI in *Cx3cr1*^GFP/+^*Ccr2*^RFP/+^ mice, which express fluorescent reporters labeling CX3CR1^+^ resident microglia/macrophages and CCR2^+^ peripheral macrophages. CCI-induced extravasated fibrinogen was co-localized with recruited macrophages and activated, amoeboid microglia, but the total number of perilesional CX3CR1^+^ microglia remained unchanged (Fig. 2A). Fibrin(ogen) deposits were also positively correlated with areas of increased axonal damage (Fig. 2B), consistent with prior findings that fibrin-mediated activation of microglia and macrophages is associated with neurotoxicity [9, 12].

Fibrin activates CNS innate immunity via the CD11b/CD18 integrin receptor expressed by microglia and monocytes [8, 9, 12]. To test the requirement for that interaction in TBI-induced neuroinflammation, we next performed CCI on transgenic *Fgg*^*γ390–396A*^ knock-in mice, in which the fibrin interaction with CD11b/CD18 is inhibited without affecting blood coagulation [7, 8]. *Fgg*^*γ390–396A*^ mice had fewer peri-lesional CD45^hi^CD11b^+^ myeloid cells at 1 and 7 days after injury (Fig. 2C), implicating a role for fibrin signaling in post-TBI inflammation.

### Fibrin-CD11b interaction induces microglia and myeloid cell oxidative stress and pro-inflammatory gene programs in TBI

**Fig. 3:**
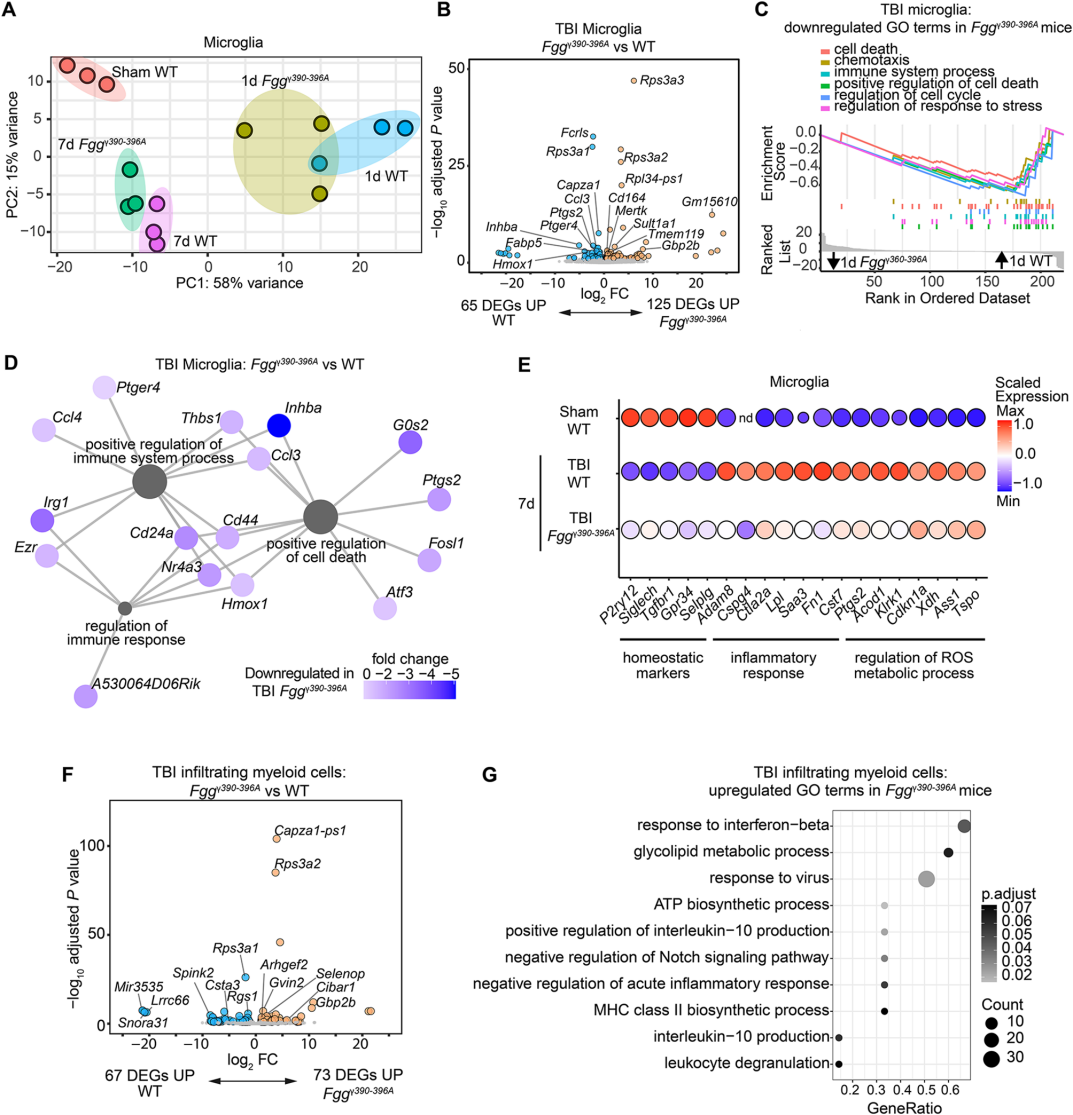
Fibrin–CD11b interaction regulates innate immune programs in TBI. (**A**) PCA analysis of RNA-seq analysis of microglia from *Fgg*^*γ390–396A*^, WT, and sham mice. 1 day (1d) after injury; 7 days (7d) after injury. Data are from *n* = 3 independent samples per group. (**B**) Volcano plot of DEGs from RNA-seq analysis of sorted microglia from *Fgg*^*γ390–396A*^ and WT mice 1d after injury. Comparison between DEGs in microglia from 1d *Fgg*^*γ390–396A*^ vs. 1d WT mice is shown. Dots depict average log_2_ fold change (FC) and -log_10_ adjusted *P* values by significance cutoff (abs(log_2_FC) > 1, adjusted *P* value < 0.1 with Wald test followed by Benjamini-Hochberg multiple test correction). Top DEGs are shown. Data are from *n* = 3 independent samples per group. (**C, D**) GSEA plots (C) or coexpression networks (D) of downregulated gene ontology (GO) terms in 1d *Fgg*^*γ390–396A*^ vs. 1d WT microglia. (**E**) Dot plot of selected gene markers expressed by microglia from sham, 7d WT, and 7d *Fgg*^*γ390–396A*^ mice. Data are from *n* = 3 independent samples per group. nd, not detected. (**F**) Volcano plot of DEGs from RNA-seq analysis of sorted infiltrating myeloid cells from 1d *Fgg*^*γ390–396A*^ and 1d WT mice. Dots depict average log_2_FC and -log_10_ adjusted *P* values by significance cutoff (abs(log_2_FC) > 1, adjusted *P* value < 0.1 with Wald test followed by Benjamini-Hochberg multiple test correction). Top DEGs are shown. Data are from *n* = 3 independent samples per group. (**G**) GSEA dot plot of GO terms significantly upregulated in infiltrating myeloid cells from 1d *Fgg*^*γ390–396A*^ vs. 1d WT mice

Next, we performed RNA-seq of peri-lesional CD45^lo^CD11b^+^ microglia and CD45^hi^CD11b^+^ infiltrating myeloid cells from *Fgg*^*γ390–396A*^ and WT mice at 1 and 7 d following CCI. Differential gene expression and unbiased gene set enrichment analysis (GSEA) at 1 d post-CCI showed robust transcriptional changes, including upregulation of genes related to ‘inflammatory response’, ‘MAPK cascade’, and ‘regulation of ROS metabolic process’ in WT mice compared to sham controls (Fig. 3A and Fig. S1A, B). In contrast to those in WT mice, microglia from *Fgg*^*γ390–396A*^ mice showed distinct differentially expressed genes (DEGs) in response to TBI, with decreased inflammatory DEGs (i.e., *Hmox1*, *Ptgs2*, *Ccl3*) and increased homeostatic gene markers (i.e., *P2ry12*, *Tmem119*, *Mertk*) (Fig. 3B and Fig. S1A, B). Microglia from *Fgg*^*γ390–396A*^ mice also had reduced expression of gene pathways regulating immune response, cell death, and chemotaxis compared to WT mice (Fig. 3C, D). These data suggest that fibrin signaling via CD11b/CD18 serves as an early activator of pro-oxidative microglial responses in TBI. Interestingly, DEGs involved in the inflammatory response and reactive oxygen species metabolic pathways remained elevated in microglia from WT mice at 7 days following TBI. These changes were largely reduced in *Fgg*^*γ390–396A*^ mice, suggesting a role for fibrin in regulating sustained polarization of pathogenic microglia after injury (Fig. 3E). Similar to microglia, infiltrating myeloid cells from *Fgg*^*γ390–396A*^ mice showed greater expression of genes linked to suppression of the acute inflammatory response and interleukin-10 production compared to myeloid cells from WT mice (Fig. 3F, G). Together, these findings suggest that fibrin-CD11b interaction promotes prooxidant and proinflammatory gene expression in innate immune cells after TBI.

### Targeting fibrin-CD11b interaction protects TBI mice from oxidative stress and neuronal injury

**Fig. 4:**
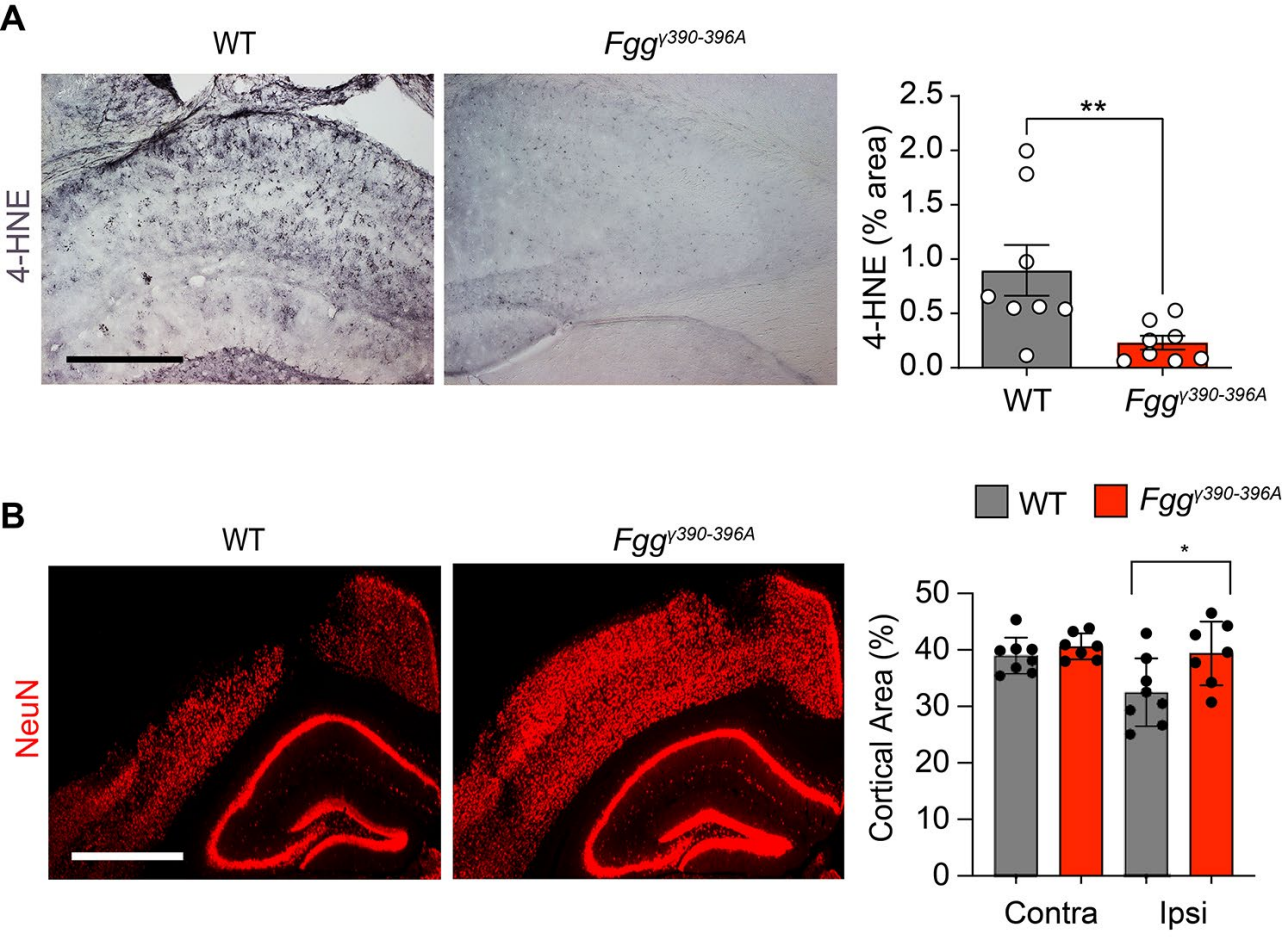
Blocking fibrin–CD11b interactions protects mice against TBI-related oxidative stress and neuronal injury. (**A, B**) Representative brain sections from 7d WT and 7d *Fgg*^*γ390–396A*^ mice stained for 4-HNE (A) or NeuN (B). Scale bars, 0.5 mm (A), 1 mm (B). Image quantification is shown. Data are presented as mean ± s.e.m.; *n* = 7–8 mice per group (1 mouse per point). *P < 0.05, ** *P* < 0.01 by two-tailed Mann-Whitney test (A) and two-way ANOVA with Bonferroni’s multiple comparisons test (B)

Given that these transcriptional changes are consistent with oxidative stress and neurotoxic inflammation [12], we tested whether *Fgg*^*γ390–396A*^ mice are protected from TBI-induced pathology. Indeed, oxidative damage and cortical loss were both significantly reduced in *Fgg*^*γ390–396A*^ male mice compared to WT mice (Fig. 4A, B) at 7 days following injury. These results are consistent with studies in *Fgg*^*γ390–396A*^ mice showing reduced oxidative stress in animal models of MS, AD and periodontitis [10–13, 29], as well as with the pro-oxidant fibrin transcriptomic and phosphoproteomic signature in innate immune cells [12, 30], suggesting that in diseases with vascular damage or increased vascular permeability, fibrinogen is an apical signal orchestrating oxidative stress responses [30, 31]. Collectively, these results suggest that fibrin signaling induces inflammatory and oxidative stress gene signatures in microglia and macrophages contributing to neuropathology in TBI.

## Discussion

Historically, the clinical significance of fibrinogen in TBI has primarily been related to its clotting function in the hyperacute period (e.g. TBI-induced coagulopathy, intracranial hemorrhage) [32–36]. Here, we report a role for fibrinogen in TBI-induced neuroinflammation. We show that in both human patient samples and a mouse model of TBI, fibrin deposition co-localizes with activated microglia and peripheral myeloid cells in the injured cortex. This association between fibrin and activation of innate immunity is similar to that seen in other neurological diseases (e.g. stroke, MS, AD) [26, 37–40]. Uncovering a link between extravasated fibrinogen and induction of neuroinflammation would provide a mechanism by which TBI-induced BBB dysfunction leads to long-term neurologic consequences, including the potential for development of AD [41, 42]. Indeed, it is widely accepted that TBI increases the risk for dementia [43, 44], although whether the pathogenic features of post-traumatic dementia precisely recapitulate those of idiopathic AD is a matter of debate [45]. Regardless, an improved understanding of the neurovascular and immune consequences of TBI may lead to novel approaches to prevent this outcome.

We identified multiple mechanisms by which fibrin promotes brain inflammation in TBI. Interaction between the fibrinogen γ chain and the CD11b/CD18 receptor facilitates the recruitment of myeloid cells in the acute (1d) post-TBI period and promotes transition of microglia from a homeostatic state to oxidative stress and neurodegenerative phenotype. These findings are in accordance with prior studies showing a role for fibrin–CD11b/CD18 signaling in blood-induced innate immune polarization and subsequent neurotoxic microglial programming in MS and AD [11, 12]. Furthermore, our findings suggest that fibrin-CD11b signaling is associated with a pro-inflammatory response in infiltrating myeloid cells, similar to its association with microglial activation. These sequelae are likely responsible for TBI-induced oxidative damage and cortical loss observed in our study, and provides an underlying mechanism triggering monocyte and microglia post-TBI neurotoxicity [16, 46–49].

Integrating these mechanistic links into TBI discourse enhances our understanding of its underlying pathophysiological processes and lays the groundwork for future research in TBI-induced neuroinflammation. The pathway of BBB breakdown, fibrin extravasation, and subsequent induction of an innate immune response presented here appears to be shared among neurologic diseases [6, 12, 31]. This mechanism also presents a therapeutic target [11], which holds particular interest in TBI, where there is a near complete absence of therapies [50]. However, the potential for therapeutic intervention in TBI must be viewed within the context of the deleterious and beneficial effects of inflammation after neurotrauma [51, 52]. Indeed, broadly dampening the immune response acutely after injury is ill-advised [53] and future studies will be required to determine the optimal therapeutic window for interventions targeting fibrin-induced inflammation. A potential advantage of selectively targeting fibrin may be suppression of neurodegenerative genes without global inactivation of the innate immune response [12]. Fibrin-CD11b signaling induces a specific signature in microglia and macrophages, activating oxidative stress and neurodegenerative genes [12]. Like in the current study in the context of TBI, genetic elimination of the fibrin inflammatory domain in AD mice selectively inhibits oxidative stress and neurodegenerative genes accompanied by restoration of the microglia homeostatic signatures [12, 54]. Inhibition of fibrin-CD11b signaling also improves clinical outcomes including a reduction in cognitive impairment and protection from paralysis in AD and MS mouse models, respectively [8, 10, 11]. Supporting this, treatment with a fibrin-specific monoclonal antibody, which targets the fibrin inflammatory domain γ377–395 epitope, is protective in animal models of AD and MS by selectively blocking fibrin-induced activation of microglia and macrophages, without affecting innate immune cell activation by other stimuli, such as LPS [11]. Given the similarities in selective suppression of neurotoxic signatures in TBI (this study), AD and MS mouse models [8, 10, 11], it is possible that fibrin blockade might also be beneficial for recovery after TBI.

A risk of targeting fibrin in TBI is the potential for adverse effects on coagulation, which would exacerbate intracranial hemorrhage [11, 31]. Studies in *Fgg*^*γ390–396A*^ mice have shown that targeting the cryptic γ377–395 epitope of fibrin does not adversely affect coagulation [7]. The *Fgg*^*γ390–396A*^ mice maintain normal fibrinogen concentrations, fibrin polymerization, platelet aggregation, and thrombus formation, as well as normal hemostatic responses even after vascular injury, BBB disruption in autoimmune models, periodontitis, or hemorrhagic colitis [8, 29, 55, 56]. Furthermore, *Fgg*^*γ390–396A*^ mice do not develop spontaneous hemorrhagic events and tolerate major surgeries similar to WT mice without excessive bleeding, in stark contrast to fibrinogen knock-out mice, which lack fibrinogen and exhibit broad hemostatic abnormalities [7]. This suggests an opportunity whereby pathologic fibrin-induced inflammation can be specifically targeted without risking a hemorrhagic complication [11, 31]. Supporting this, a fibrin-targeting immunotherapy against the γ377–395 epitope is therapeutic without adverse effects on blood coagulation [11]. Given the ability to selectively inhibit fibrinogen’s inflammatory properties without impacting its clotting functions, we posit that targeting the γ377–395 epitope could be a therapeutic strategy to reduce pathogenic inflammation in post-TBI settings, warranting further pre-clinical investigations in neurotrauma.

In evaluating the limitations of our study, we first acknowledge that there are several differences between mouse models and human TBI. The CCI model we use shows a month-long decline in fibrinogen extravasation. However, the pathological severity of TBI combined with impaired fibrinolytic mechanisms in neurologic conditions may contribute to prolonged fibrin(ogen) deposition in human TBI [57, 58]. Our histological analyses in mice focused on fibrinogen distribution and immune cell counts localized to the cortical contusion. As TBI often produces multiple pathologies throughout the brain, including fibrin extravasation [5], we anticipate that future studies dedicated to detecting the full extent of cellular changes following neurotrauma using unbiased stereology may shed further light on the role of fibrin in propagating TBI-induced inflammation beyond the peri-lesional cortex (e.g., white matter injury) in additional time points using larger animal cohorts. Moreover, comprehensive human neuropathological studies are required for understanding the spatial and temporal correlation of fibrin(ogen) deposition with immune activation in human TBI. Furthermore, given the sex differences of microglia in neuroinflammation [59–61], additional studies will be required to assess the role of the BBB and fibrinogen in female mice post-TBI. Understanding sex-specific immune responses will further provide insights into TBI pathophysiology and its long-term consequences, including the potential development of dementia [42]. While our study focused on the direct effects of fibrin in TBI, the role of fibrinolysis products in CNS inflammation does warrant attention [62]. The enzymatic breakdown of fibrin clots during fibrinolysis produces various degradation products, such as D-dimer and other fibrin fragments [6, 40, 62]. These fibrinolysis products also contribute to CNS inflammation by activating microglia and attracting peripheral immune cells to the injury site. This process could intensify the inflammatory milieu within the CNS, potentially exacerbating neuronal damage, oxidative injury and impacting post-TBI recovery. Therefore, understanding the interplay between fibrin deposition and fibrinolysis may identify potential therapeutic targets within the fibrinolysis pathway to alleviate neuroinflammation, oxidative damage and support neural recovery [63, 64].

## Conclusions

In summary, we found in humans and mice that TBI induces both acute and chronic BBB disruption with persistent fibrin deposition that is associated with CNS innate immune activation. Our data shed new light on the emerging role of the inflammatory fibrin epitope in regulating neurotoxic innate immunity by revealing a causal mechanism by which the fibrin- interaction with CD11b/CD18 mediates oxidative damage and cortical loss following TBI. Our study indicates that fibrin-CD11b signaling induces neurotoxic microglial and monocyte gene expression that may potentially influence inflammation and oxidative stress in the injured brain. Selective targeting of fibrin proinflammatory signaling may be a valuable approach for a disease currently lacking in targeted therapies.

### Electronic supplementary material

Below is the link to the electronic supplementary material.

**Supplemental Video 1**. TBI induces widespread fibrin(ogen) deposition. Three-dimensional reconstruction of mouse brain 2-days after CCI labeled with Alexa^647^-fibrinogen (red) and 2MDa Dextran-casted vessels (green). Scale bar, 1 mm. Data are representative of *n* = 3 mice.

**Supplementary Figure 1**. RNA-seq analysis of CNS innate immune cells in TBI. **(A)** Volcano plot of DEGs from RNA-seq analysis of sorted microglia from 1d WT mice or sham control mice. Dots depict average log_2_ fold change (FC) and -log_10_ adjusted *P* values by significance cutoff (abs(log_2_FC) > 1.5, adjusted *P* value < 0.05 with Wald test followed by Benjamini-Hochberg multiple test correction). Top DEGs are shown. Data are from *n* = 3 independent samples per group. (**B**) GSEA plots of upregulated GO terms in microglia from 1d WT microglia vs. sham mice.

## Data Availability

Genome Expression Omnibus (GEO) data supporting the findings of this study have been deposited in the GEO depository under accession number GSE253476. All other data are available in the manuscript or the supplementary materials. Any additional data can be made available from the corresponding author upon request.

## Abbreviations

TBI: Traumatic brain injury
BBB: Blood-brain barrier
CCI: Controlled cortical impact
ROS: Reactive oxygen species
AD: Alzheimer’s disease
MS: Multiple sclerosis
ROI: Region of interest
RNA-seq: RNA sequencing
GSEA: Gene set enrichment analysis
DEGs: Differentially expressed genes
GO: Gene ontology
4-HNE: 4-hydroxynoneal
