# Supplementary figures and images for: Fibrin promotes oxidative stress and neuronal loss in traumatic brain injury via innate immune activation
Source: J Neuroinflammation. 2024 Apr 15;21:94. doi: 10.1186/s12974-024-03092-w (PMC11017541; doi:10.1186/s12974-024-03092-w)

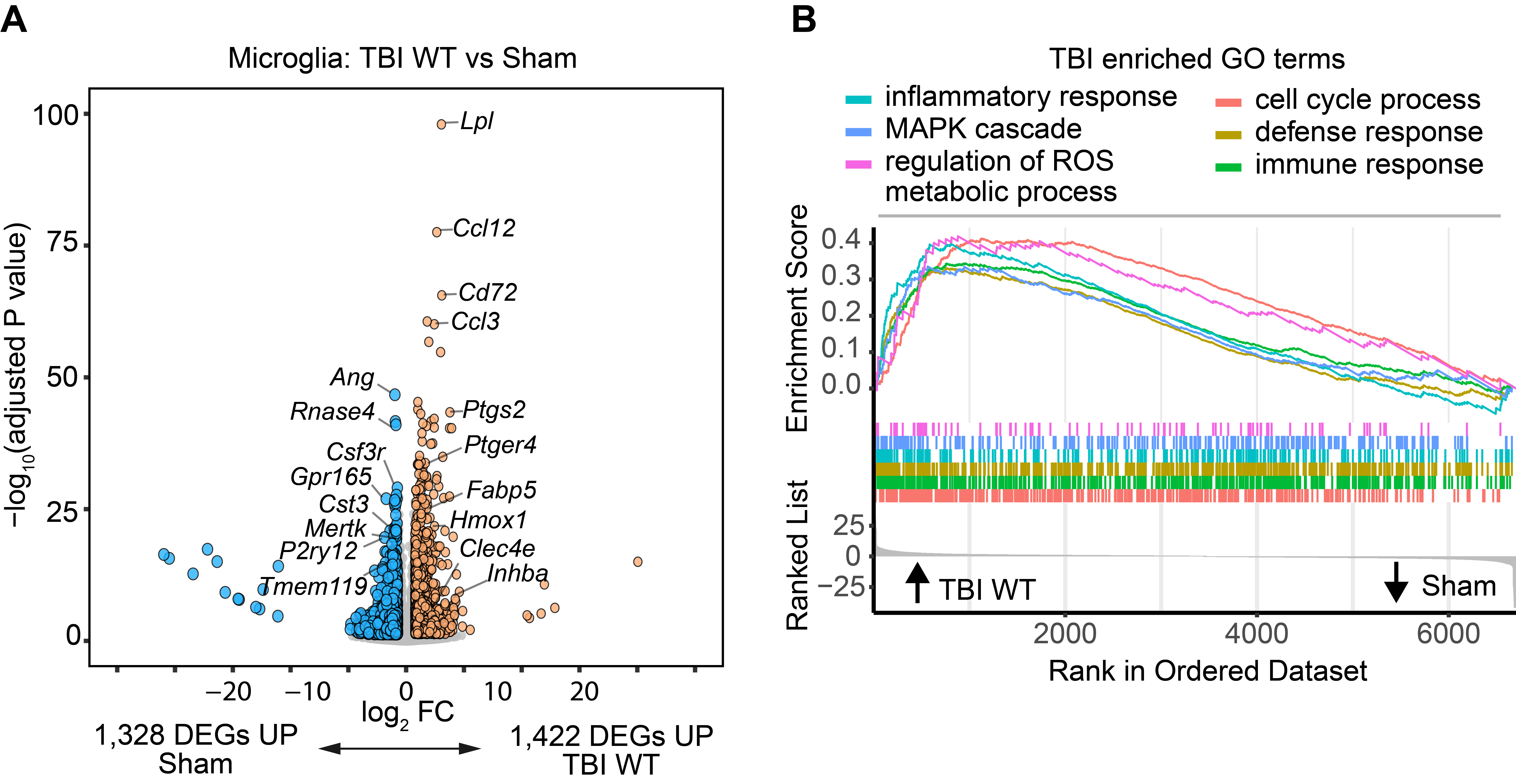

Supplement: Supplementary file 2 — Supplementary Figure 1. RNA-seq analysis of CNS innate immune cells in TBI. (A) Volcano plot of DEGs from RNA-seq analysis of sorted microglia from 1d WT mice or sham control mice. Dots depict average log2 fold change (FC) and -log10 adjusted P values by significance cutoff (abs(log2FC) > 1.5, adjusted P value < 0.05 with Wald test followed by Benjamini-Hochberg multiple test correction). Top DEGs are shown. Data are from n = 3 independent samples per group. (B) GSEA plots of upregulated GO terms in microglia from 1d WT microglia vs. sham mice. [file 12974_2024_3092_MOESM2_ESM.jpg]
